# Relationship between glycated hemoglobin levels and three-month outcomes in acute ischemic stroke patients with or without diabetes: a prospective Korean cohort study

**DOI:** 10.1186/s12883-024-03581-8

**Published:** 2024-03-04

**Authors:** Changchun Cao, Tony Bowei Wang, Haofei Hu, Yong Han, Xiaohua Zhang, Yulong Wang

**Affiliations:** 1 Department of Rehabilitation, Shenzhen Dapeng New District Nan’ao People’s Hospital, No. 6, Renmin Road, Dapeng New District, Shenzhen, 518000 Guangdong Province China; 2 Skidmore College, 815 North Broadway, Mailbox 8411, Saratoga Spring, NY 12866-1632 USA; 3 Department of Nephrology, Futian District, Shenzhen Second People’s Hospital, The First Affiliated Hospital of Shenzhen University, No.3002, Sungang West Road, Shenzhen, 518000 Guangdong Province China; 4 Department of Emergency, Shenzhen Second People’s Hospital, The First Affiliated Hospital of Shenzhen University, Shenzhen, 518000 Guangdong Province China; 5 Department of Rehabilitation, Futian District, Shenzhen Second People’s Hospital, The First Affiliated Hospital of Shenzhen University, No.3002, Sungang West Road, Shenzhen, 518000 Guangdong Province China

**Keywords:** Glycated hemoglobin, Acute ischemic stroke, Adverse clinical outcomes, Nonlinear association, Generalized additive model, Smooth curve fitting

## Abstract

**Objective:**

In patients experiencing acute ischemic stroke, there is ongoing debate surrounding the connection between chronic hyperglycemic status and their initial clinical outcomes. Our objective was to examine the connection between glycated hemoglobin (HbA1c) levels and adverse clinical outcomes at both 3-months adverse clinical outcomes in individuals with acute ischemic stroke (AIS) with and without diabetes.

**Methods:**

The present prospective cohort study involved 896 AIS patients without diabetes and 628 with diabetes treated at a South Korean hospital from January 2010 to December 2016. The target independent variable is HbA1c. The outcome variable is a modified Rankin scale score ≥ 3. A binary logistic regression model was applied to assess the connection between HbA1c levels and 3-month poor clinical outcomes in AIS patients with and without diabetes. Additionally, a generalized additive model and smoothed curve fitting were utilized to explore potential nonlinear associations between HbA1c levels and 3-month adverse clinical outcomes in AIS patients with and without diabetes.

**Results:**

The binary logistic regression model could not identify any statistically significant connection between HbA1c and 3-month adverse clinical outcomes in AIS patients, both those with and without diabetes, after correcting for various factors. However, a nonlinear relationship emerged between HbA1c and 3-month adverse clinical outcomes in AIS patients with diabetes. The inflection point for HbA1c was determined to be 6.1%. For HbA1c values ≤ 6.1%, an inverse association was observed between HbA1c and 3-month adverse clinical outcomes in diabetic AIS patients, and each 1% increase in HbA1c in AIS patients with DM was associated with an 87% reduction in 3-month adverse clinical outcomes (OR = 0.13, 95% CI: 0.02–0.81). Conversely, when HbA1c exceeded 6.1%, a positive association between HbA1c and 3-month adverse clinical outcomes became apparent in diabetic AIS patients, and each 1% increase in HbA1c in AIS patients with DM was associated with a 23% increase in 3-month adverse clinical outcomes (OR = 1.23, 95%CI: 1.03–1.47). However, it’s important to note that no significant linear or nonlinear relationships were observed between HbA1c levels and 3-month adverse clinical outcomes in AIS patients without diabetes.

**Conclusion:**

Our findings suggest a nonlinear connection and threshold effect between HbA1c and 3-month adverse clinical outcomes in AIS patients with diabetes. AIS patients with diabetes had a lower risk of 3-month adverse clinical outcomes when their HbA1c control was close to 6.1%. Our findings may aid treatment decision-making and potentially guide interventions to optimize glycemic control in AIS patients.

**Supplementary Information:**

The online version contains supplementary material available at 10.1186/s12883-024-03581-8.

## Introduction

Acute ischemic stroke (AIS) contributes substantially to human disability and mortality, imposing a significant economic and public health burden [1–3]. Despite optimal medical interventions, 40% of acute ischemic stroke patients experience poor prognoses, underscoring the need to identify factors influencing their outcomes [4, 5] actively. Glycemic control has emerged as a pivotal determinant in the prognosis of AIS patients, with mounting evidence linking glycemic dysregulation to adverse outcomes.

Glycated hemoglobin (HbA1c), a marker reflecting average glycemia levels over the preceding 2–3 months, indicates previous glucose control or chronic hyperglycemia [4, 5]. Elevated HbA1c levels are associated with an increased risk of cardiovascular diseases and mortality [6, 7]. Yet, the prognostic significance of HbA1c in the context of functional outcomes following AIS engenders debate within the scientific community. While extant research posits that HbA1c may act as an independent indicator of functional prognosis post-AIS, transcending the presence or absence of diabetes mellitus (DM) [8, 9], the evidence is replete with contradictory findings. Some studies affirm the correlation between increased HbA1c concentrations and adverse functional prognoses, specifically in diabetic AIS populations, while others discern no such correlation in individuals devoid of diabetes [10–12]. In light of these inconsistencies, using published data from a prospective study in Korea, this study explores the potential association between HbA1c levels and 3-month adverse clinical outcomes in patients with AIS with and without diabetes.

## Methods

### Study design

This cohort study utilized data collected between January 2010 and December 2016 from a single-center prospective registry system in South Korea [13]. The study focused on the 3-month adverse clinical outcomes as the dependent variable and HbA1c as the independent variable in AIS patients.

### Data source

Kang MK et al. [14] generously provided the study's raw data without cost. The study has been published under an open-access framework, utilizing a Creative Commons Attribution license. This license permits unrestricted usage, distribution, and reproduction in any format as long as due credit is given to the original author and source. We express our gratitude to the data contributors for their invaluable contributions.

### Study population

The initial investigators assembled patients with AIS who were admitted to the hospital within seven days of symptom onset [13]. This was accomplished using data from a prospective registry based in a single center [13]. The original research received approval from the institutional review board of Seoul National University Hospital, which also granted a waiver for patient consent (IRB NO. 1009–062-332) [13]. As a result, ethical clearance was not deemed necessary for this subsequent analysis. Furthermore, the original study adhered to the principles outlined in the Declaration of Helsinki, with all procedures conducted in alignment with relevant standards and regulations, as outlined in the Declarations section. The same applies to this secondary analysis.

**Fig. 1:**
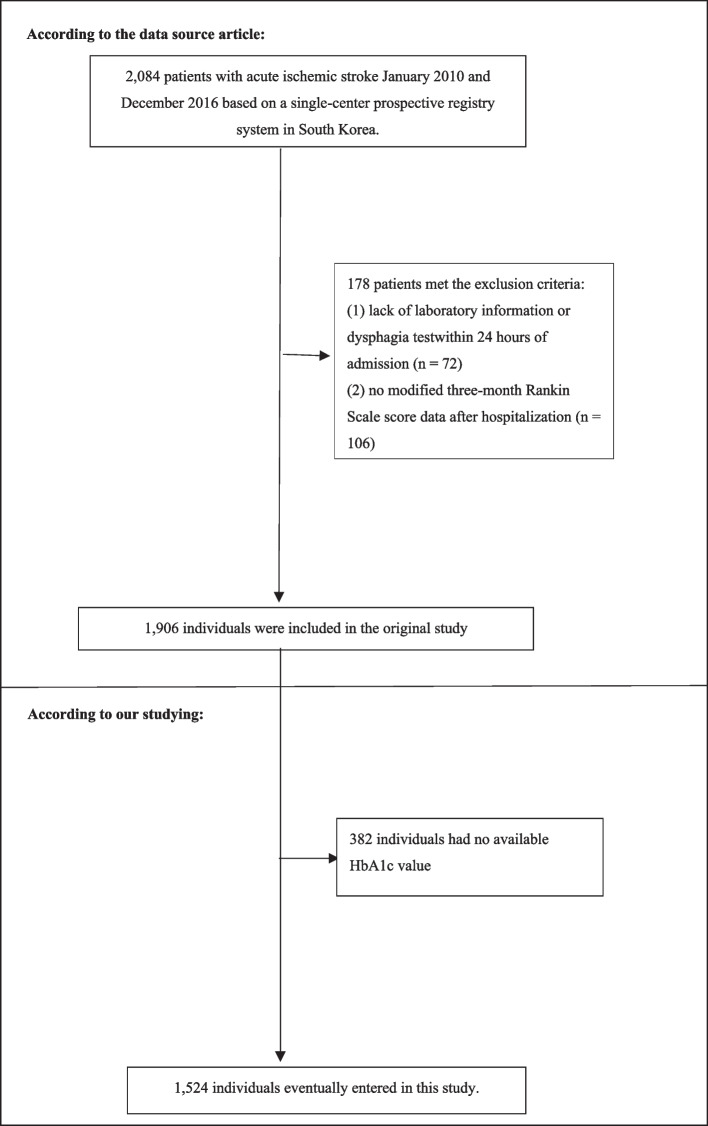
Flowchart of study participants

The initial research involved 2,084 individuals diagnosed with AIS. The exclusion of 178 participants was based on exclusion criteria in the initial study [13]. These criteria consisted of (1) absence of dysphagia test or laboratory information within 24 h of admission (*n* = 72); (2) absence of a 3-month modified Rankin Scale (mRS) score following hospitalization (*n* = 106). Consequently, a cohort of 1,906 people was included in the analysis of the original research. This study did not consider participants who presented with missing HbA1c values (*n* = 382). As a result, the current investigation involved a total of 1,524 individuals diagnosed with AIS. The process of participant selection is visually represented in Fig. 1.

### Variables

#### HbA1c

HbA1c was noted as a continuous variable. HbA1c is tested within 24 h of admission and collected from electronic medical records [13].

#### DM

DM was defined as an HbA1c level of at least 6.5% [14] or a diagnosis from an electronic medical record system [13].

#### Three-month adverse clinical outcomes in participants with AIS

The mRS score, which measures disability/dependence and death (score 6) after a stroke, was used to evaluate outcomes at the 3-month adverse clinical outcomes following the onset of AIS. If patients with AIS died within 3 months after hospitalization, their mRS score in the 3rd month was 6 points. Data was collected via telephone or structured outpatient interviews. The participants were divided into two categories: the adverse clinical outcomes group and the good clinical outcomes group. Adverse clinical outcomes were defined as mRS scores ≥ 3.

#### Covariates

Covariates were selected based on prior research findings and our clinical expertise. The covariates utilized included the following: (1) Categorical variables: previous stroke/transient ischemic attack (TIA), sex, coronary heart disease (CHD), diabetes mellitus (DM), smoking status, age, stroke etiology, and hypertension. (2) Continuous variables: total cholesterol (TC), hemoglobin concentration (HGB), triglycerides (TG), serum albumin (ALB), high-density lipoprotein cholesterol (HDL-C), C-reactive protein, serum creatinine (Scr), low-density lipoprotein cholesterol (LDL-C), and the National Institutes of Health Stroke Scale (NIHSS) score. Laboratory information is tested within 24 h of admission and collected from electronic medical records [13].

#### Data collection

Data collection details were sourced from the original study [13]. Using an automatic scale, skilled nurses measured patients’ height and weight upon admission. Weight was measured on an under-the-bed scale, and height with a tape measure for patients with severe stroke who could not stand on their own. Laboratory data, such as TG, HGB, ALB, LDL-C, C-reactive protein, HDL-C, and Scr, were extracted from electronic medical records. The NIHSS score was evaluated upon admission to assess initial neurological severity.

#### Missing data handling

In our study, there were missing data for several variables as follows: C-reactive protein had 207 cases (13.583%), LDL-C had 14 cases (0.919%), TG had 47 cases (3.084%), TC had 1 case (0.066%), and HDL-C had 40 cases (2.625%). We employed multiple imputations during the study's modeling phase to address these missing values and mitigate the potential impact of incomplete variables on our analysis [15, 16]. The imputation model incorporated age, smoking status, HDL-C, ALB, sex, TG, BMI, HGB, C-reactive protein, LDL-C, Scr, TC, previous stroke or TIA, DM, stroke etiology, CHD, hypertension, and NIHSS score. Linear regression was utilized with ten iterations as the imputation model's type. The missing data analysis followed the Missing-at-Random (MAR) assumptions [16].

### Statistical analysis

All participants were divided into two groups based on the presence of diabetes. For non-diabetics, we divided them into three groups based on HbA1c (HbA1c < 5.5%, 5.5% ≤ HbA1c < 6%, 6 ≤ HbA1c < 6.5%). For diabetic patients, we divided them into four groups according to HbA1c (HbA1c < 6%, 6 ≤ HbA1c < 6.5%, 6.5 ≤ HbA1c < 7% and HbA1c ≥ 7%). The continuous baseline data were expressed as the mean ± the standard deviation (SD) (normally distributed data) and medians (quartile) (nonnormally distributed data). The current research used the expression in numbers (percentages) for categorical data. Comparisons were made using either ANOVA (normally distributed data), the χ2 test (categorical data), or the Kruskal–Wallis test (skewed data). Incidence rates were expressed in cumulative incidence.

After conducting covariate screening, we developed three distinct models utilizing univariate and multivariate binary logistic regression approaches. These models aimed to investigate the correlation between HbA1c and 3-month adverse clinical outcomes in AIS patients with and without diabetes. The models were structured as follows: (1) An unadjusted model (Model 1), where no covariates were adjusted; (2) A minimally-adjusted model (Model 2), which accounted for adjusting for smoking status, sex, BMI, and age; (3) A fully-adjusted model (Model 3), which accounted for age, smoking status, sex, TG, BMI, HGB, HDL-C, ALB, C-reactive protein, LDL-C, Scr, previous stroke or TIA, CHD, hypertension, stroke etiology and NIHSS score. We calculated effect sizes with 95% confidence intervals (95%CI). Confounding variables were adjusted based on clinical knowledge, literature reports, and univariate analysis results [17, 18]. Additionally, TC was excluded from the final multivariate logistic regression equation due to collinearity with other factors (Table S1).

Generalized additive models (GAM) and smooth curve fitting through penalized splines were employed to scrutinize the nonlinear association between HbA1c and 3-month adverse clinical outcomes in AIS patients with or without diabetes. Upon identifying a nonlinear relationship, a recursive algorithm was employed to determine the inflection point. This algorithm began with random initialization and then utilized filtering/smoothing to locate the inflection point. Subsequently, a two-piece binary logistic regression model was constructed on both sides of this point. The log-likelihood ratio test helps to select the optimal model to describe the association between HbA1c and 3-month adverse clinical outcomes in AIS participants.

We bolstered our findings with various sensitivity analyses. HbA1c was transformed into a categorical variable as per HbA1c quartiles. We assessed the P trend to validate results for HbA1c as a continuous variable and explore potential nonlinear associations. Sensitivity analyses excluded CHD and smoking status; they are strongly connected to 3-month adverse clinical outcomes in AIS patients [19, 20].

In accordance with the STROBE guidelines, we adhered to rigorous reporting standards for all findings [21]. Statistical analyses were conducted using Empower Stats, and R Statistical significance was defined as a *P* value of 0.05 (two-sided).

## Results

### Characteristics of participants

Table 1 outlines the demographic and clinical attributes of the study participants. The final analysis encompassed 1,524 individuals, with 60.7% being men. Among them, 363 (23.8%) were below 60 years of age, 408 (26.8%) were between 60 and 70 years of age, 520 (34.1%) were between 70 and 80 years of age and 233 (15.3%) were above 80 years of age.

**Table 1:** The baseline characteristics of participants

HbA1c	Non-DM			*P*-value	DM				*P*-value
	HbA1c (< 5.5%)	HbA1c (5.5%-6.0%)	HbA1c (6.0%-6.5%)		HbA1c (< 6.0%)	HbA1c (6.0%-6.5%)	HbA1c (6.5%-7.0%)	HbA1c (≥ 7.0%)	
Participants	158	502	236		60	90	181	297	
Gender				0.232					0.073
Male	94 (59.5%)	322 (64.1%)	137 (58.1%)		42 (70.0%)	44 (48.9%)	109 (60.2%)	177 (59.6%)	
Female	64 (40.5%)	180 (35.9%)	99 (41.9%)		18 (30.0%)	46 (51.1%)	72 (39.8%)	120 (40.4%)	
Age(years)				< 0.001					0.354
< 60	80 (50.6%)	121 (24.1%)	44 (18.6%)		12 (20.0%)	16 (17.8%)	26 (14.4%)	64 (21.5%)	
60 to < 70	26 (16.5%)	140 (27.9%)	60 (25.4%)		14 (23.3%)	25 (27.8%)	51 (28.2%)	92 (31.0%)	
70 to < 80	38 (24.1%)	161 (32.1%)	89 (37.7%)		23 (38.3%)	30 (33.3%)	75 (41.4%)	104 (35.0%)	
≥ 80	14 (8.9%)	80 (15.9%)	43 (18.2%)		11 (18.3%)	19 (21.1%)	29 (16.0%)	37 (12.5%)	
BMI (kg/m^2^)	22.8 ± 3.1	23.5 ± 3.0	23.5 ± 3.2	0.034	22.6 ± 3.2	23.1 ± 2.9	23.9 ± 3.2	24.4 ± 3.5	< 0.001
Smoking status				0.772					0.330
No	93 (58.9%)	302 (60.2%)	147 (62.3%)		34 (56.7%)	61 (67.8%)	115 (63.5%)	174 (58.6%)	
Yes	65 (41.1%)	200 (39.8%)	89 (37.7%)		26 (43.3%)	29 (32.2%)	66 (36.5%)	123 (41.4%)	
NIHSS score	3 (1–7)	3(1-)	3 (1–6)	0.937	4.5 (0–23)	4 (0–28)	3 (0–25)	4 (0–30)	0.358
Hypertension				0.009					0.001
No	86 (54.4%)	214 (42.6%)	93 (39.4%)		6 (10.0%)	12 (13.3%)	50 (27.6%)	83 (27.9%)	
Yes	72 (45.6%)	288 (57.4%)	143 (60.6%)		54 (90.0%)	78 (86.7%)	131 (72.4%)	214 (72.1%)	
CHD				0.453					0.226
No	146 (92.4%)	453 (90.2%)	209 (88.6%)		47 (78.3%)	80 (88.9%)	150 (82.9%)	257 (86.5%)	
Yes	12 (7.6%)	49 (9.8%)	27 (11.4%)		13 (21.7%)	10 (11.1%)	31 (17.1%)	40 (13.5%)	
Previous stroke/TIA				0.249					0.336
No	134 (84.8%)	412 (82.1%)	185 (78.4%)		45 (75.0%)	63 (70.0%)	137 (75.7%)	235 (79.1%)	
Yes	24 (15.2%)	90 (17.9%)	51 (21.6%)		15 (25.0%)	27 (30.0%)	44 (24.3%)	62 (20.9%)	
Stroke etiology				0.179					0.405
SVO	38 (24.1%)	145 (28.9%)	67 (28.4%)		17 (28.3%)	32 (35.6%)	67 (37.0%)	121 (40.7%)	
LAA	27 (17.1%)	93 (18.5%)	43 (18.2%)		11 (18.3%)	17 (18.9%)	44 (24.3%)	70 (23.6%)	
CE	41 (25.9%)	139 (27.7%)	78 (33.1%)		19 (31.7%)	24 (26.7%)	39 (21.5%)	50 (16.8%)	
Other determined	23 (14.6%)	47 (9.4%)	24 (10.2%)		4 (6.7%)	7 (7.8%)	8 (4.4%)	19 (6.4%)	
Undetermined	29 (18.4%)	78 (15.5%)	24 (10.2%)		9 (15.0%)	10 (11.1%)	23 (12.7%)	37 (12.5%)	
Death				0.973					0.373
No	152 (96.2%)	481 (95.8%)	226 (95.8%)		56 (93.3%)	88 (97.8%)	171 (94.5%)	287 (96.6%)	
Yes	6 (3.8%)	21 (4.2%)	10 (4.2%)		4 (6.7%)	2 (2.2%)	10 (5.5%)	10 (3.4%)	
TC (mg/dL)	182.2 ± 38.1	184.3 ± 44.2	185.5 ± 44.6	0.757	164.7 ± 43.7	166.3 ± 37.6	174.1 ± 42.2	179.2 ± 49.1	0.031
TG (mg/dL)	107.2 ± 60.3	106.3 ± 49.7	112.1 ± 56.4	0.379	99.0 ± 51.7	121.0 ± 64.8	118.3 ± 58.3	129.4 ± 67.5	0.006
HDL-C(mg/dL)	47.8 ± 14.8	47.8 ± 13.9	47.3 ± 14.0	0.898	48.0 ± 15.1	44.9 ± 15.7	44.0 ± 12.5	42.9 ± 13.0	0.058
LDL-C (mg/dL)	107.9 ± 33.9	112.2 ± 37.9	114.1 ± 39.9	0.274	95.8 ± 39.5	94.9 ± 30.1	104.0 ± 35.8	106.2 ± 40.3	0.036
ALB (g/dL)	4.1 ± 0.4	4.1 ± 0.4	4.0 ± 0.4	0.098	3.9 ± 0.4	4.0 ± 0.5	4.0 ± 0.4	4.0 ± 0.5	0.332
Scr (mg/dL)	0.9 ± 0.6	1.0 ± 0.9	1.0 ± 0.4	0.740	2.1 ± 2.4	1.3 ± 1.3	1.2 ± 1.2	1.1 ± 1.0	< 0.001
HGB (g/dL)	13.8 ± 2.1	13.8 ± 1.8	13.5 ± 1.9	0.083	12.6 ± 1.9	12.6 ± 2.3	13.4 ± 1.9	13.5 ± 2.0	< 0.001
C-reactive protein (mg/dL)	0.9 ± 2.7	0.8 ± 2.1	1.2 ± 3.3	0.224	1.7 ± 3.0	0.8 ± 2.5	1.1 ± 3.1	1.4 ± 3.9	0.306
HbA1c (%)	5.3 ± 0.2	5.7 ± 0.1	6.2 ± 0.1	< 0.001	5.7 ± 0.2	6.2 ± 0.1	6.7 ± 0.1	8.3 ± 1.2	< 0.001

Values are n (%) or mean ± SD or median (quartile)

*DM* Diabetes mellitus, *HGB* Hemoglobin concentration, *BMI* Body mass index, *TC* Total cholesterol, *TG* Triglyceride, *LDL-C* Low-density lipoproteins cholesterol, *HDL-C* High-density lipoprotein cholesterol, *Scr* Serum creatinine, *ALB* Serum albumin, *BMI* Body mass index, *CHD* Coronary heart disease, *HbA1c* Glycated hemoglobin, *TIA* Transient ischemia attack, *NIHSS* National institute of health stroke scale, *SVO* Small vessel occlusion, *LAA* Small vessel occlusion, *CE* Cardio embolism

We categorized patients into subgroups based on the presence of diabetes and HbA1c. Among the non-diabetic patients, compared to the low level HbA1c group (HbA1c < 5.5% and 5.5% ≤ HbA1c < 6.0%), the highest level of HbA1c group (6.0% ≤ HbA1c < 6.5%) had the highest levels of age and BMI and the highest proportion of hypertension. Among the diabetic patients, the lowest level of HbA1c group (HbA1c < 6.0%) had the highest levels of Scr and C-reactive protein and the highest proportion of hypertension. Additionally, compared to the high level HbA1c group (6.0% ≤ HbA1c < 6.5%, 6.5% ≤ HbA1c < 7.0%, and HbA1c ≥ 7.0), the lowest level of HbA1c group (HbA1c < 6.0%) among the diabetic patients had the lowest levels of TC, TG, LDL-C, HGB, and BMI.

### The cumulative incidence of 3-month adverse clinical outcomes in AIS patients

Table 2 shows that 431 participants experienced 3-month adverse clinical outcomes. The overall cumulative incidence of 3-month adverse clinical outcomes in AIS patients without DM was 24.6%. Specifically for AIS patients without DM, the cumulative incidence of 3-month adverse clinical outcomes was 21.5%, 25.1%, and 25.4% in the HbA1c < 5.5% group, 5.5% ≤ HbA1c < 6.0% group, and 6.0% ≤ HbA1c < 6.5% group, respectively. Furthermore, the overall cumulative incidence of unfavorable outcomes among AIS patients with DM was 33.6%. Among AIS patients with DM, the cumulative incidence prevalence of unfavorable outcomes was 45.0%, 30.0%, 33.7%, and 32.3% in the HbA1c < 6.0% group, 6.0% ≤ HbA1c < 6.5% group, 6.5% ≤ HbA1c < 7.0% group, and HbA1c ≥ 7.0 group, respectively. The lowest incidence of 3-month adverse clinical outcomes was found in patients without DM in the HbA1c < 5.5% group and patients with DM in the 6.0% ≤ HbA1c < 6.5% group, respectively.

**Table 2:** Cumulative incidence of 3-month adverse clinical outcomes in AIS patients with and without diabetes

HbA1c (%)	Participants	Participants with adverse clinical outcomes	Cumulative incidence (95% CI) (%)
**Non-DM**			
Total	896	220	24.6 (21.7–27.4)
HbA1c (< 5.5%)	158	34	21.5 (15.0–28.0)
HbA1c (5.5%-6.0%)	502	126	25.1 (21.3–28.9)
HbA1c (6.0%-6.5%)	236	60	25.4 (19.8–31.0)
**DM**			
Total	628	211	33.6 (29.9–37.3)
HbA1c (< 6.0%)	60	27	45.0 (32.0–58.0)
HbA1c (6.0%-6.5%)	90	27	30.0 (20.3–39.7)
HbA1c (6.5%-7.0%)	181	61	33.7 (26.7–40.7)
HbA1c (≥ 7.0%)	297	96	32.3 (27.0–37.7)

*HbA1c* Glycated hemoglobin, *AIS* Acute ischemic stroke, *DM* Diabetes mellitus, *CI* Confidence

### Results of multivariate analysis using binary logistic regression models

Based on binary logistic regression models, Table 3 shows the connection between HbA1c and 3-month adverse clinical outcomes in AIS patients. This study used three models to scrutinize the potential correlation between HbA1c and 3-month adverse clinical outcomes in AIS patients with and without diabetes. In Model 1, for each 1% increase in HbA1c, the association with the development of 3-month adverse clinical outcomes in AIS patients was not statistically significant for both nondiabetic and diabetic cohorts (Non-DM group, OR = 1.42, 95%CI: 0.88–2.27 *P* = 0.149; DM group, OR = 1.01, 95%CI: 0.89–1.15 *P* = 0.836). In addition, our results based on the binary logistic regression model also showed no statistically significant association between HbA1c and 3-month adverse clinical outcomes in Model 2 and Model 3 (*P* > 0.05).

**Table 3:** Relationship between HbA1c and 3-month adverse clinical outcomes in AIS patients with and without diabetes

Exposure		Model 1 (OR,95%CI) P	Model 2 (OR,95%CI) P	Model 3 (OR,95%CI) P
Non-DM	HbA1c (per 1% increase)	1.42 (0.88, 2.27) 0.149	1.23 (0.75, 2.03) 0.419	1.68 (0.92, 3.07) 0.092
	HbA1c group			
	HbA1c (< 5.5%)	0.82 (0.53, 1.26) 0.360	0.84 (0.53, 1.33) 0.449	0.84 (0.49, 1.46) 0.542
	HbA1c (5.5%-6.0%)	Ref	Ref	Ref
	HbA1c (6.0%-6.5%)	1.02 (0.71, 1.45) 0.925	0.94 (0.65, 1.36) 0.733	1.03 (0.67, 1.60) 0.888
DM	HbA1c (per 1% increase)	1.01 (0.89, 1.15) 0.836	1.06 (0.93, 1.21) 0.374	1.14 (0.97, 1.35) 0.118
	HbA1c group			
	HbA1c (< 6.0%)	1.91 (0.97, 3.77) 0.062	2.17 (1.07, 4.40) 0.032	2.27 (0.94, 5.50) 0.069
	HbA1c (6.0%-6.5%)	Ref	Ref	Ref
	HbA1c (6.5%-7.0%)	1.19 (0.69, 2.05) 0.540	1.32 (0.75, 2.33) 0.338	1.92 (0.95, 3.86) 0.069
	HbA1c (≥ 7.0%)	1.11 (0.67, 1.86) 0.679	1.35 (0.79, 2.31) 0.276	1.70 (0.88, 3.31) 0.116

Model 1: we did not adjust other covariates

Model 2: we adjusted smoking status, sex, BMI, and age

Model 3: we adjusted age, smoking status, sex, TG, BMI, HGB, HDL-C, ALB, C-reactive protein, LDL-C, Scr, previous stroke or TIA, CHD, hypertension, stroke etiology, and NIHSS score

*HbA1c* Glycated hemoglobin, *AIS* Acute ischemic stroke, *DM* Diabetes mellitus, *OR* Odds ratios, *CI* Confidence

Besides, we converted HbA1c from a continuous variable to a categorical variable and reintroduced the categorically transformed HbA1c into the model. The findings from Model 3 revealed that among AIS patients without diabetes, the OR for the HbA1c < 5.5% group and 6.0% ≤ HbA1c < 6.5% group were 0.84 (95%CI: 0.49–1.46) and 1.03 (95%CI: 0.67–1.60), respectively, when compared to the reference group (5.5% ≤ HbA1c < 6.0%) of HbA1c. Similarly, among AIS patients with diabetes, the OR for the HbA1c < 6.0% group, 6.5% ≤ HbA1c < 7.0% group, and HbA1c ≥ 7.0 group were 2.27 (95%CI: 0.94–5.50), 1.92 (95%CI: 0.95–3.86), and 1.70 (95%CI: 0.88–3.31), respectively, also in reference to the 6.0% ≤ HbA1c < 6.5% group. The confidence interval distribution showed no statistically significant relationship between HbA1c (categorical) derived from the model and 3-month adverse clinical outcomes in AIS patients.

### The nonlinearity addressed by the generalized additive model

**Fig. 2:**
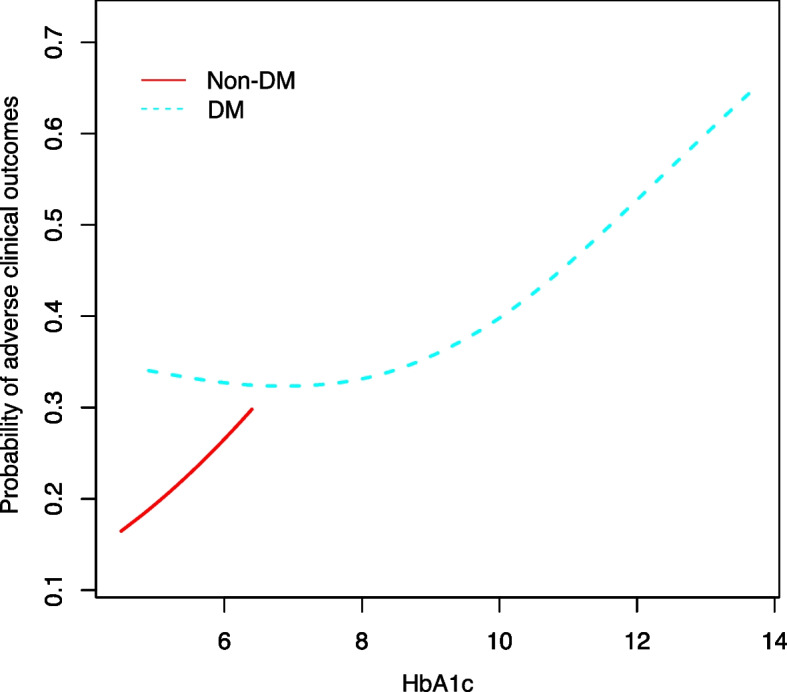
The nonlinear relationship between HbA1c and 3-month adverse clinical outcomes in AIS patients with and without diabetes. A nonlinear relationship was detected in AIS patients with diabetes after adjusting for age, smoking status, sex, TG, BMI, HGB, HDL-C, ALB, C-reactive protein, LDL-C, Scr, previous stroke or TIA, CHD, hypertension, stroke etiology, and NIHSS score

We further explored whether there was a nonlinear association between HbA1c and 3-month adverse clinical outcomes in AIS patients with or without DM. We discovered a nonlinear relationship between HbA1c and 3-month adverse clinical outcomes in AIS patients with DM using the GAM and smooth curve fitting (adjusting for age, smoking status, sex, TG, BMI, HGB, HDL-C, ALB, C-reactive protein, LDL-C, Scr, previous stroke or TIA, CHD, hypertension, stroke etiology, and NIHSS score) (Fig. 2). However, we were unable to establish a nonlinear relationship between HbA1c and 3-month adverse clinical outcomes in AIS patients without DM. By employing a recursive algorithm, we first determined the HbA1c inflection point, which was 6.1 in AIS patients with DM. Then, the effect sizes and confidence intervals on both sides of the inflection point were then assessed using a two-piecewise binary logistic regression model. On the right side of the inflection point, each 1% increase in HbA1c in AIS patients with DM was associated with a 23% increase in 3-month adverse clinical outcomes (OR = 1.23, 95%CI: 1.03–1.47). However, the effect size (OR) to the left of the inflection point was 0.13 (95%CI: 0.02–0.81) (Table 4), and each 1% increase in HbA1c was associated with an 87% reduction in 3-month adverse clinical outcomes.

**Table 4:** The results of a two-piecewise binary logistic regression model

3-month adverse clinical outcomes	All participants (OR,95%CI, P)	Non-DM (OR,95%CI, P)	DM (OR,95%CI, P)
Fitting model by standard linear regression (per 1% increase for HbA1c)	1.12 (0.97, 1.30) 0.129	1.68 (0.92, 3.07) 0.092	1.14 (0.97, 1.35) 0.118
Fitting model by two-piecewise binary logistic regression			
Inflection point of HbA1c (%)	8.2	5.4	6.1
≤ Inflection point (per 1% increase for HbA1c)	0.98 (0.76, 1.26) 0.866	0.69 (0.06, 7.96) 0.766	0.13 (0.02, 0.81) 0.029
> Inflection point (per 1% increase for HbA1c)	1.33 (0.99, 1.79) 0.060	1.942 (0.94, 4.0) 0.072	1.23 (1.03, 1.47) 0.021
P for log-likelihood ratio test	0.197	0.476	0.018

Note 1: In all participants, we adjusted age, smoking status, sex, TG, BMI, HGB, HDL-C, ALB, C-reactive protein, LDL-C, Scr, previous stroke or TIA, CHD, DM, hypertension, and NIHSS score

Note 2: For DM and Non-DM subgroups, we adjusted for age, smoking status, sex, TG, BMI, HGB, HDL-C, ALB, C-reactive protein, LDL-C, Scr, previous stroke or TIA, CHD, hypertension, stroke etiology, and NIHSS score

*HbA1c* Glycated hemoglobin, *OR* Odds ratios, *CI* Confidence, *DM* Diabetes mellitus

### Sensitivity analysis

We conducted sensitivity analyses focused on participants without CHD, and thorough adjustments were made for potential confounding variables, including age, smoking status, sex, TG, BMI, HGB, HDL-C, ALB, C-reactive protein, LDL-C, Scr, previous stroke or TIA, hypertension, stroke etiology, and NIHSS score. The outcomes underscored a nonlinear relationship between HbA1c and 3-month adverse clinical outcomes in AIS patients with DM. In contrast, this connection did not hold linearly or nonlinearly for AIS patients without DM.

Moreover, we extended our scrutiny to exclude smoking participants. Following analogous adjustments for the array of confounding variables, akin outcomes emerged. Specifically, there was a nonlinear correlation between HbA1c and 3-month adverse clinical outcomes in AIS patients with DM, while the relationship remained statistically insignificant for AIS patients without DM (Table 5). Collectively, based on the comprehensive span of sensitivity analyses undertaken, there is some robustness to the results of our study.

**Table 5:** Relationship between HbA1c and 3-month adverse clinical outcomes in AIS patients with and without diabetes analyzed by two-piecewise binary logistic regression model in different sensitivity analyses

3-month adverse clinical outcomes	Non-DM (OR,95%CI, P)	DM (OR,95%CI, P)	Total (OR,95%CI, P)
Model 4			
Fitting model by two-piecewise binary logistic regression			
Inflection point of HbA1c (%)	5.9	5.9	8.3
≤ Inflection point (per 1% increase for HbA1c)	1.33 (0.51, 3.50) 0.559	0.02 (0.00, 0.73) 0.032	0.99 (0.76, 1.30) 0.943
> Inflection point (per 1% increase for HbA1c)	3.36 (0.64, 17.77) 0.154	1.22 (1.02, 1.46) 0.032	1.36 (0.98, 1.87) 0.063
P for log-likelihood ratio test	0.425	0.018	0.217
Model 5			
Fitting model by two-piecewise binary logistic regression			
Inflection point of HbA1c (%)	5.7	6.0	8.3
≤ Inflection point (per 1% increase for HbA1c)	4.26 (0.68, 26.66) 0.122	0.02 (0.00, 0.48) 0.015	0.89 (0.64, 1.23) 0.481
> Inflection point (per 1% increase for HbA1c)	1.490 (0.381, 5.833) 0.5665	1.24 (0.99, 1.55) 0.058	1.61 (1.09, 2.37) 0.016
P for log-likelihood ratio test	0.447	0.007	0.050

Model 4 was a sensitivity analysis in participants without CHD. We adjusted age, smoking status, sex, TG, BMI, HGB, HDL-C, ALB, C-reactive protein, LDL-C, Scr, previous stroke or TIA, hypertension, stroke etiology, and NIHSS score

Model 5 was a sensitivity analysis conducted on non-smoking participants. We adjusted age, sex, TG, BMI, HGB, HDL-C, ALB, C-reactive protein, LDL-C, Scr, previous stroke or TIA, CHD, hypertension, stroke etiology, and NIHSS score

*HbA1c* Glycated hemoglobin, *OR* Odds ratios, *CI* Confidence, *DM* Diabetes mellitus

## Discussion

The current study's main goal was to investigate the relationship between HbA1c and 3-month adverse clinical outcomes for AIS patients with and without DM. Our research found a nonlinear relationship between HbA1c and adverse clinical outcomes in AIS patients with DM. In addition, we found that HbA1c among AIS patients with DM had a threshold impact, with a clear change happening at a precise inflection point of 6.1%. Conversely, no significant linear or nonlinear associations between HbA1c levels and 3-month adverse clinical outcomes were seen in AIS patients without DM.

While many studies have shown a link between HbA1c and cardiovascular diseases and mortality, research on the connection between HbA1c and clinical outcomes in AIS patients is controversial. A retrospective study of 526 people with diabetes and 1351 people without diabetes found that elevated HbA1c was associated with poorer 3-month outcomes in ischemic stroke patients with and without diabetes after adjusting for age, sex, hypertension, NIHSS score, systolic and diastolic blood pressure, and blood glucose, stroke subtype and stroke-related complications [8]. In a retrospective study of 408 patients with first acute ischemic stroke, after adjusting for age, sex, intravenous recombinant tissue plasminogen activator, NIHSS score, admission glucose, TG, stroke subtype, hypertension, atrial fibrillation, smoking, uric acid, systolic blood pressure, and diastolic blood pressure, the results showed that HbA1c was correlated with 3-month adverse clinical outcomes of AIS patients with DM (OR: 1.482, 95%CI: 1.013–2.167), while there was no correlation HbA1c and between 3-month adverse clinical outcomes of patients without DM (OR:1.355, 95%CI: 0.589–3.118) [10]. In a multicenter retrospective study involving 484 patients with acute ischemic stroke, HbA1c was not associated with poor neurologic outcomes in AIS patients with and without DM after adjusting for age, sex, NIHSS at admission, atrial fibrillation, and fasting glucose (OR: 1.700, 95%CI: 0.494–5.859 for patients without DM, OR: 1.163, 95%CI: 0.945–1.430 for patients with DM) [12]. In addition, another retrospective study involving 267 patients with acute minor ischemic stroke treated with intravenous thrombolysis found that HbA1c was not associated with 90-day functional outcome after adjusting for age, sex, NIHSS on admission, fibrinogen, hypersensitive C-reactive protein, fasting glucose, DM, stroke subtype, and early neurological deterioration (OR: 0.975, 95%CI: 0.747–1.271) [22]. However, the results of this research based on the binary logistic regression model are inconsistent with the results of the above studies. For this result, we speculate that a nonlinear relationship may exist between HbA1c and 3-month adverse clinical outcomes. Therefore, we used GAM and smooth curve fitting to validate the nonlinear association between HbA1c and 3-month adverse clinical outcomes. In our present research, this finding indicates a nonlinear relationship and threshold effect of HbA1c and 3-month adverse clinical outcomes in AIS patients with DM. The following possible explanations cause these inconsistent results: (1) the study population differed; (2) many studies with those various conclusions failed to explain the nonlinear link effectively; (3) in comparison with our study, those studies did not consider the effect of ALB, Scr, and CHD on the connection between HbA1c and 3-month adverse clinical outcomes when controlling for confounding factors. However, earlier research suggested that ALB, Scr, and CHD were closely linked to adverse clinical outcomes [20, 23, 24]; (4) differences in HbA1c levels at baseline may also play a role.

Earlier investigations did not investigate the potential curvilinear link between HbA1c and adverse clinical outcomes for AIS patients. For the first time, we analyzed the nonlinear connection between HbA1c and 3-month adverse clinical outcomes for AIS patients with and without DM in this current research. The result of the smooth curve fitting and GAM showed that the connection between HbA1c and 3-month adverse clinical outcomes for AIS patients with DM was nonlinear after controlling for confounders. We utilized a two-piecewise binary logistic regression model regression model to determine the inflection point of HbA1c. When HbA1c levels in diabetic patients were ≤ 6.1%, a 1% increase in HbA1c levels was associated with an 87% decrease in 3-month adverse clinical outcomes. However, when HbA1c levels in diabetic patients were > 6.1%, a 1% increase in HbA1c levels was associated with a 23% increase in 3-month adverse clinical outcomes. In the diabetic group, AIS patients with HbA1c < 6.0% had the highest levels of Scr, C-reactive protein, the lowest HGB, and the highest percentage of hypertension. These are risk factors for adverse outcomes in AIS patients [18, 24, 25]. Therefore, in order to reduce the risk of adverse clinical outcomes, HbA1c should ideally be controlled at around 6.1% in diabetic patients with AIS.

However, the underlying factors driving the nonlinear relationship between HbA1c and adverse clinical outcomes among AIS patients remain unclear. Tight glycaemic control may lead to hypoglycaemic episodes [26]. Several studies have shown that hypoglycemia is strongly associated with cardiovascular events, increased risk of mortality, and poor prognosis for stroke [26–28]. Prolonged hypoglycemia could indicate malnutrition and impact neural repair processes [29]. Given the metabolic stress and heightened energy demands associated with AIS, factors such as post-stroke neuroendocrine sympathetic activation, cytokine release, and anaerobic free radical buildup can create an imbalance between catabolic and anabolic processes, necessitating increased energy expenditure [30]. Consequently, sustained hypoglycemia could detrimentally affect early neurological repair in AIS patients. However, when blood glucose keeps rising, persistent hyperglycemia might contribute to an expanded infarct volume, leading to a poorer prognosis [31]. There is evidence that a pro-oxidative condition brought on by hyperglycemia might cause direct neurotoxicity [32]. Moreover, oxidative stress and heightened coagulation factors triggered by prolonged hyperglycemia could increase the risk of thrombosis, particularly anterior thrombosis [33]. This may explain that in patients with diabetes mellitus, either persistent hypoglycaemic or prolonged hyperglycaemic state increases the poor clinical outcome in stroke patients.

Our study boasts several strengths. Primarily, we delved into the intricate nonlinear relationship between HbA1c and adverse clinical outcomes among AIS patients. Rigorous statistical adjustments were applied to mitigate residual confounding, lending robustness to our findings. Moreover, a battery of sensitivity analyses was conducted to bolster the reliability of our results. These encompassed categorizing HbA1c, incorporating continuous covariates into the analysis using GAM, and re-evaluating the HbA1c-adverse outcome association after excluding participants with CHD or smoking history.

Nonetheless, the study does carry some limitations. First, the study cohort primarily comprises Koreans, necessitating further validation of these findings in diverse racial groups. Second, some variables suffered from data gaps. For instance, the initial dataset employed age stratification in 10 intervals rather than patient-specific ages, potentially leading to incomplete variable information. Third, the absence of multiple HbA1c measurements during the follow-up period constitutes another limitation. Fourth, as is common in observational research, unmeasured or uncontrollable confounders, such as intravenous thrombolysis, and a detailed medication history (the type, dose, and duration of antidiabetic medication), cannot be entirely ruled out despite meticulous control over recognized potential confounders. Fifth, there may be selection bias in our study. We excluded 106 patients with missing 3-month mRS data, considering that these patients may have been lost to follow-up. Our study had a loss-to-follow-up rate of approximately 5%. Previous studies have shown that a loss to follow-up rate of 5% or less usually results in a small bias [34]. Sixth, the sample size within certain subsets of our study, specifically the 6.0% ≤ HbA1c < 6.5% group in diabetes, is indeed limited. Seventh, conditions that affect red blood cell turnover (hemolytic and other anemias, glucose-6-phosphate dehydrogenase deficiency, recent blood transfusion, use of drugs that stimulate erythropoiesis, end-stage kidney disease, and pregnancy), ethnic populations and hemoglobin variants may result in discrepancies between the HbA1C result and the patient's true mean glycemia [35]. We should use the combination of results from continuous glucose monitoring (CGM)/blood glucose monitoring (BGM) and HbA1c to evaluate glycemic control. In the future, we can consider designing our studies with a larger and more diverse sample size to collect as many variables as possible, including information on HbA1c during follow-up and CGM/BGM, intravenous thrombolysis, age, and detailed medication history (the type, dose, and duration of antidiabetic medication).

## Conclusion

This current research demonstrated a nonlinear relationship and threshold effect between HbA1c and 3-month adverse clinical outcomes in AIS patients with diabetes in Korea. However, we could not establish a nonlinear relationship between HbA1c and 3-month adverse clinical outcomes in AIS patients without DM. This finding is useful for clinicians as it provides a reference point for controlling blood glucose in AIS patients.

### Supplementary Information

**Supplementary Materials 1. **

## Data Availability

The “PLoS One” database allows for the downloading of data (https://journals.plos.org/plosone/article?id=10.1371/journal.pone.0228738).

## Abbreviations

BMI: Body mass index
HGB: Hemoglobin concentration
AIS: Acute ischemic stroke
TC: Total cholesterol
HDL-C: High-density lipoprotein cholesterol
LDL-C: Low-density lipoproteins cholesterol
TG: Triglyceride
Scr: Serum creatinine
TIA: Transient ischemia attack
HBA1c: Glycated hemoglobin
ALB: Serum albumin
CHD: Coronary heart disease
DM: Diabetes mellitus
NIHSS: National Institutes of Health Stroke Scale
GAM: Generalized additive models
mRS: Modified Rankin Scale
OR: Odds ratio
CI: Confidence interval
