# Supplementary material for: Relationship between glycated hemoglobin levels and three-month outcomes in acute ischemic stroke patients with or without diabetes: a prospective Korean cohort study
Source: BMC Neurol. 2024 Mar 4;24:85. doi: 10.1186/s12883-024-03581-8 (PMC10910674; doi:10.1186/s12883-024-03581-8)
Supplement: Supplementary file 1 — Supplementary Materials 1. [file 12883_2024_3581_MOESM1_ESM.docx]

**Table S1** The results of the collinearity screening

|  | Step 1 | Step 2 |
| --- | --- | --- |
| Gender | 1.8 | 1.8 |
| Age | 1.2 | 1.2 |
| BMI | 1.2 | 1.2 |
| Smoking status | 1.5 | 1.5 |
| NIHSS score | 1.1 | 1.1 |
| Hypertension | 1.2 | 1.2 |
| CHD | 1.1 | 1.1 |
| Previous stroke/TIA | 1 | 1 |
| DM | 1.1 | 1.1 |
| Stroke etiology | 1.1 | 1.1 |
| TC | 6.7 | NA |
| TG | 1.5 | 1.3 |
| HDL-C | 1.6 | 1.3 |
| LDL-C | 5.4 | 1.2 |
| ALB | 1.6 | 1.6 |
| Scr | 1.2 | 1.2 |
| HGB | 1.8 | 1.8 |
| C-reactive protein | 1.3 | 1.2 |

HGB, hemoglobin concentration; BMI, body mass index; TC, total cholesterol; TG, triglyceride; LDL-C, low-density lipoproteins cholesterol; HDL-C, high-density lipoprotein cholesterol; Scr, serum creatinine; ALB, serum albumin; BMI, body mass index; CHD, coronary heart disease; TIA, transient ischemia attack; NIHSS, national institute of health stroke scale; DM, diabetes mellitus.
